# Exploring the beliefs and experiences of older Irish adults and family carers during the novel coronavirus (COVID-19) pandemic: A qualitative study protocol

**DOI:** 10.12688/hrbopenres.13031.1

**Published:** 2020-04-20

**Authors:** Katie Robinson, Aoife O'Neill, Mairead Conneely, AnnMarie Morrissey, Siobhan Leahy, Pauline Meskell, Judi Pettigrew, Rose Galvin

**Affiliations:** 1 School of Allied Health, University of Limerick, Limerick, V94 T9PX, Ireland; 2 Ageing Research Centre, Health Research Institute, University of Limerick, Limerick, V94 T9PX, Ireland; 3 Department of Nursing and Midwifery, University of Limerick, Limerick, V94 T9PX, Ireland

**Keywords:** COVID-19, Qualitative Research, Older adults, Public and Patient Involvement

## Abstract

**Background**: In December 2019 a novel human coronavirus (COVID-19) was identified in Wuhan, China (Wu et al, 2020). The virus subsequently spread to most countries worldwide and the World Health Organisation characterised the outbreak a pandemic on March 11 ^th^ 2020 (WHO, 2020a). Older age is associated with an increased risk of mortality in patients with COVID-19 (Chen et al., 2020). In March 2020, the Irish Government introduced 'cocooning' as a measure for those over 70 years of age to minimise interactions with others by not leaving their homes (Dept. of Health, 2020). The COVID-19 pandemic presents unique threats to the health and well-being of older adults. This study aims to explore the longitudinal experiences and beliefs of older adults during the COVID-19 pandemic. Findings will be important for tailoring supports, interventions and public health information for this population.

**Methods**: A longitudinal exploratory qualitative study will be conducted using repeated semi-structured telephone interviews with a convenient sample of older adults recruited from participants of an older adult and family carer stakeholder panel for health services research established by the Ageing Research Centre (ARC) at the University of Limerick and through known older adult contacts of ARC academic members. Interviews will be audio recorded, transcribed and analysed using a reflexive approach to thematic analysis. Participants will have the opportunity to review and discuss preliminary analysis of the interview data and to co-write / design dissemination materials.

**Ethics and Dissemination**: Ethical approval has been granted by the Faculty of Education and Health Sciences University of Limerick, Research Ethics Committee (2020_03_51_EHS (ER)). Findings will be disseminated through open access journal publications and distribution of lay summaries, a press release and an infographic to organisations of and for older people in Ireland, broadcast and print media.

## Introduction

As of March 11 ^th^, 2020, the WHO declared the novel human coronavirus (COVID-19) outbreak a pandemic ( WHO, 2020a). There are currently (April 3 ^rd^, 2020) almost one million cases globally and 3849 confirmed cases in Ireland ( WHO, 2020b).

Three main profiles of the clinical course of COVID-19 have been reported: mild illness with upper respiratory tract symptoms, non-life-threatening pneumonia, and severe pneumonia with acute respiratory distress syndrome ( WHO 2020, cited by Heymann *et al*., 2020). A summary of findings from the largest case series to date of COVID-19 in China (72314 cases, updated through February 11, 2020) reported most cases (81%) were classified as mild, 14% were severe, and 5% were critical. In this case series study an overall case-fatality rate (CFR) of 2.3% was identified. However, those aged 70 to 79 years had an 8.0% CFR and those aged 80 years+ had a 14.8% CFR ( Wu & McGoogan, 2020). Furthermore, older people with multimorbidity, frailty and underlying conditions such as hypertension, respiratory system disease, and cardiovascular disease are at higher risk of severe disease if infected with COVID-19 ( Garnier-Crussard *et al*., 2020; Kunz & Minder, 2020; Yang *et al*., 2020). Older adults are also vulnerable due to age-related immune system changes ( Weyand & Goronzy, 2016).

To date there has been no effective treatment of COVID-19 and no vaccine is currently available ( Lai *et al*., 2020). Public health measures have included a focus on limiting human-to-human transmission in order to reduce secondary infections and infection control interventions, including avoiding close contact with people with respiratory symptoms, hand hygiene, cough etiquette ( Lai *et al*., 2020; WHO, 2019). The Irish government have implemented a range of measures, including closure of non-essential services, travel restrictions and for people over 70 years on March 27 ^th^ the government introduced ‘cocooning’ to minimise interactions by older adults, with others, by not leaving their homes ( Department of Health, 2020).

The COVID-19 pandemic is the third coronavirus outbreak in the past two decades. The severe acute respiratory syndrome (SARS) epidemic in 2003 infected over 8,000 persons and caused 774 deaths ( Peiris *et al*., 2003). Middle East respiratory syndrome (MERS) was first reported in 2012 and is still circulating with a total of 2494 confirmed cases at the end of November 2019 and 858 associated deaths ( WHO, 2019). Research on SARS and MERS coronavirus outbreaks is relevant in considering the experiences of people during the COVID-19 pandemic although COVID-19 cases far outstrip both other viruses. Prior research on older adults’ experiences, beliefs and behaviours during the SARS outbreak is concerning. Gaps in knowledge and poor uptake of precautionary behaviours during the SARS outbreak were reported in a survey of older adults in Hong Kong (n=112) ( So *et al*., 2004). For example, only 58% reported they ‘very often’ covered their mouth when sneezing/coughing ( So *et al*., 2004). Similarly, a telephone interview study of psychosocial factors that influenced the practice of preventive behaviours against SARS among older Chinese adults (n=354) found only 40.7% of the older participants practiced the recommended SARS preventive behaviours ( Tang & Wong, 2005). Adoption of preventative behaviours was associated with; perceived vulnerability to SARS, self-efficacy, and confidence in local health authorities ( Tang & Wong, 2005). Age-related differences in emotional responses and coping were also identified during SARS. A study at the peak and the end of the SARS outbreak, of 385 Hong Kong Chinese, concluded that older adults responded to the SARS outbreak with less anger and were better able to alter their coping strategies in response to the changing environment than younger adults ( Yeung & Fung, 2007).

We could not locate any qualitative research on older adults’ experiences of SARS or MERS. Qualitative research offers an opportunity to study subjective experience in-depth with consideration of wider contextual factors. Qualitative studies of the experience of other infectious diseases has revealed important considerations for clinical practice and health policy. For example, qualitative research on the experience of tuberculosis has revealed that stigma can have a negative impact on individuals living with tuberculosis and may lead to withdrawal from society ( Juniarti & Evans, 2011), leading to recommendations that ensuring the confidentiality of people with tuberculosis should be a central element of tuberculosis management strategies ( Juniarti & Evans, 2011). Furthermore, qualitative ethnographic research, on the consequences of the 2001 foot and mouth disease epidemic on a rural population in North Cumbria, found that participants reported difficult experiences of distress, anguish, horror, chaos, loss of personal security, powerlessness, disruption, loss and trauma as well as recovery, endurance and sources of support. This study also revealed a gap between knowledge derived from local experience and centralised knowledge, such as organisational directives, which were perceived as not adapting to what was happening ‘on the ground’ ( Mort *et al*., 2005). Critically, Mort and colleagues emphasised that their qualitative research with people directly affected by the epidemic focused on their experiential contribution differed from the findings of official inquiries into the outbreak (2005). 

Gaining a fuller understanding of older people’s experiences during the COVID-19 pandemic is important as both infectious diseases and measures to deal with them, such as quarantine, pose unique threats to the health and well-being of older adults. A recently published rapid review of the impact of quarantine reported negative psychological effects and the review authors recommend that people who are quarantined need information, effective communication, supplies (general and medical), the quarantine period should be short, the altruistic choice of self-isolating/quarantine should be emphasised by public health officials and finally, voluntary quarantine is associated with less distress and fewer long-term complications than mandatory quarantine ( Brooks *et al*., 2020). 

Public health measures to prevent transmission increase risks of loneliness and social isolation which are both well-established risk factors for physical and mental illness in later life ( Courtin & Knapp, 2017; Ong *et al*., 2016).

The SARS epidemic in 2003 was associated with an increase in older adults' suicide rate in Hong Kong ( Cheung *et al*., 2008). Factors identified in analysis of Coroner Court reports of SARS-related cases of older adults who died by suicide included fear of contracting SARS, concerns about hygiene, obsession with SARS related news media, social isolation, disruption of social life and activities and disconnectedness ( Yip *et al*., 2010). Notwithstanding regional variation in suicide rates, it is vitally important to understand and attempt to mitigate the psychosocial impact of an infectious disease outbreak and subsequent public health measures on older adults.

The British Geriatrics Society (2020) have called for the inclusion of older people in COVID-19 research and identify that research is needed into a number of topics aligned with this protocol, such as how older people work to overcome social isolation during the COVID-19 pandemic and the use of technology to continue clinical care while maintaining social distancing.

Older adults face unique threats to their health and well-being during the COVID-19 pandemic. In this study we aim to explore the in-depth experiences and beliefs of older Irish adults during the COVID-19 pandemic through qualitative methods. Findings will have implications for health and community services and public policy.

## Method

### Design

A longitudinal qualitative interpretative design will be employed to explore, the experiences and beliefs of older Irish adults during the COVID-19 pandemic in depth. The study will be conducted and reported in line with the Consolidated Criteria for Reporting Qualitative Research (COREQ) to ensure robust conduct and reporting of report important aspects of the study including the research team, methods, context of the study, findings, analysis and interpretations ( Tong *et al*., 2007).

### Participants and recruitment

Recruitment will be conducted via a convenience sampling procedure. In 2019, the Ageing Research Centre (ARC) at the University of Limerick, Ireland established an older adult and family carer stakeholder panel for health services research ( Conneely *et al*., 2020). This stakeholder panel is guided by a partnership-focused framework ( Greenhalgh *et al*., 2019) and participants were recruited to the stakeholder panel in 2019, following principles of purposeful and snowball sampling. Recruitment activities included public advertising in community locations and in community publications, advertisement through community and patient groups, gatekeepers in relevant organisations (e.g. Age Action Ireland). For this study, older adult and family carer ARC stakeholder panel members will be contacted by telephone by second author A.O.N., firstly to cancel a scheduled meeting, A.O.N. will subsequently present them with information on this study and offer to email an information sheet (see *Extended data* ( O’Neill, 2020)). We considered posting information sheets and consent forms; however, evidence for COVID-19 transmission via paper could not be identified. A recent preprint found COVID-19 remained viable on cardboard for 24 hours ( van Doremalen *et al*., 2020). Therefore, to avoid any risk of COVID-19 transmission via printed consent forms we decided to read information and consent forms to participants during the recruitment phone call and again at the outset of the interview. Information and consent forms will additionally be emailed to participants with email access.

Information on the study will also be circulated via email to academic researcher members of ARC who will be asked to distribute to known older adult contacts (e.g. family and friends). When prospective participants express interest in participating AON will arrange a follow up phone call a minimum of 2 days later to allow time to consider participating. Recruitment will be guided by principles of saturation. Given the proposed highly heterogeneous sample, it is anticipated that in excess of 30 participants will be recruited.

### Research team roles and prior experience

We plan for interviews to be conducted, transcribed and analysed by A.O.N., M.C., A.M.M. and K.R. In case of researcher illness between scheduling and conducting interviews during this pandemic, R.G. and J.P. may also conduct scheduled interviews. R.G. and J.P. will support data analysis by engaging in critical dialogue with the wider research team.

A.O.N. is a postdoctoral researcher and statistician employed in the Ageing Research Centre at the University of Limerick. A.O.N. is a novice qualitative researcher; however, they have established relationships with the target population for this study and will be supported and supervised by K.R., a senior lecturer in occupational therapy and an experienced qualitative researcher. K.R. also has an established relationship with the prospective participants in this study. M.C. is a doctoral candidate and physiotherapist with extensive clinical experience working with older people. M.C. has completed training in qualitative and participatory research methods as part of the structured component of her PhD programme. J.P. is a senior lecturer in occupational therapy and has a PhD in anthropology. She has conducted research on mental health and psychosocial support during the “People’s war” in Nepal and is an experienced qualitative researcher. S.L. and A.M.M. are lecturers in physiotherapy and occupational therapy and have experience of supervising and conducting qualitative research. A.M.M. has attended a previous event with the Ageing Research Centre stakeholder panel. R.G. is a senior lecturer in physiotherapy and has extensive experience of conducting and supervising qualitative research focused on the experiences of older people. P.M. is a senior lecturer in Nursing and an experienced qualitative researcher and faculty member of Evidence Synthesis Ireland.

### Data collection

Two semi-structured recorded telephone interviews will be conducted with recruited participants. Interviews via telephone were selected to avoid person to person contact that may increase the likelihood of transmission of the virus. Diary keeping was considered and rejected due to concerns about placing burden on participants during potential quarantine/self-isolation.

At the outset of each interview informed consent will be confirmed orally and audio recorded digitally (consent form available as *Extended data* ( O’Neill, 2020)). Interviews will be completed 4 to 6 weeks apart. We aim for all initial interviews to be completed while cocooning guidelines are in place for older adults.

The interview schedule was developed with attention to the potential psychosocial consequences of the COVID-19 pandemic for participants. The interview schedule covers four thematic areas; participants lived experiences since the COVID-19 outbreak, participants’ beliefs about COVID-19, challenges participants have faced since the COVID-19 outbreak, what has supported participants since the COVID-19 outbreak (see *Extended data* ( O’Neill, 2020)). Interviewers will use open questions and the interview will follow up on participant’s responses rather than adhering rigidly to the schedule. The interview schedule was piloted by A.O.N. with a fellow researcher in the ARC. Information on age, gender, living situation and employment status will also be gathered at the outset of the interview.

The research team will create a log/timeline of major events in the COVID-19 pandemic such as changes in Government policy, case numbers, etc., in parallel with data collection to contextualise the data collected.

### Data analysis

A thematic approach to analysis will be undertaken broadly situated within a critical realist theoretical framework. This framework recognises that knowledge is a subjective, discursively bound and changing social construction ( Vincent & O’Mahoney, 2018). A reflexive approach to thematic analysis ( Braun *et al.*, 2018) will be undertaken which will acknowledge and consider the centrality of researcher subjectivity. Reflexivity will enable the researcher team to consider and analyse how subjective and intersubjective elements influence the research process. At the research conception stage, we considered our relationship to the topic as recommended by Finlay (2002) and considered how our relationship to the topic might change as the COVID-19 outbreak spreads and likely affects us directly or our families. This allowed us to consider our insider/outsider perspective as our own lives are currently disrupted by public health measures such as university closure and we potentially share prospective participants’ emotional responses to the pandemic. This awareness allowed us to refine the interview schedule and consider in a nuanced way how we could engage with participants and respond to their accounts with awareness of how we could influence data collection. Reflexivity will continue throughout data collection phase to allow us to identify how the emerging researcher-participant relationship shapes data collection ( Finlay, 2002). Reflexivity will continue supported by peer discussions throughout data analysis, write up and dissemination.

When coding is complete participants will be invited to review and discuss preliminary analysis of the interview data and contribute to the process of identifying themes. Participants will also be invited to co-write/design a lay summary and infographic of the findings. Group video/phone conferences or one-to-one home calls will be scheduled to enable participants to contribute to analysis.

### Ethics

The study has received ethical approval from the Faculty of Education and Health Sciences, University of Limerick, Research Ethics Committee (2020_03_51_EHS (ER)). Interviewers will ensure that all participants have time to consider the opportunity to participate in this study. The information sheet will be read to participants at the initial phone call and again at the outset of the interviews before verbal consent is obtained. Information on the following will be presented: the research team contact details and contact details for the ethics committee that approved the study, what participation will involve, how data recorded will be stored, assurances about the voluntary nature of participation and details of any identifiable information that may be reported in publications arising from the study. At the close of the interview participants will be thanked and provided with details of:

- A helpline for older people established by the organisation ALONE in collaboration with the Department of Health and the Health Service Executive ( https://alone.ie/alone-launch-a-covid-19-support-line-for-older-people-working-in-collaboration-with-the-department-of-health-and-the-hse/).
- Government of Ireland, Department of Health advice on how to protect yourself during the COVID-19 pandemic ( https://www.gov.ie/en/publication/472f64-covid-19-coronavirus-guidance-and-advice/).

### Dissemination

Findings will be disseminated through an open access journal publication and lay summaries and an infographic will be disseminated to organisations of and for older people in Ireland (Age Action, ALONE, Family Carers Ireland), press releases will be disseminated to broadcast and print media. To support rapid dissemination of the study, findings an article will be submitted to RTÉ Brainstorm, a unique partnership between RTÉ and Irish third-level institutions. This platform allows Irish academics to present opinion, analysis and features edited by RTÉ.

## Data availability

### Underlying data

No underlying data are associated with this study.

### Extended data

Open Science Framework: Exploring the beliefs and experiences of older Irish adults and family carers during the novel coronavirus (COVID-19) pandemic: A qualitative study protocol. Extended data. https://doi.org/10.17605/OSF.IO/3WUEK ( O’Neill, 2020).

The file “extended data.docx” contains the interview schedule, information sheet, and consent form.

Extended data are available under the terms of the Creative Commons Zero "No rights reserved" data waiver (CC0 1.0 Public domain dedication).
