# Supplementary material for: Exploring the beliefs and experiences of older Irish adults and family carers during the novel coronavirus (COVID-19) pandemic: A qualitative study protocol
Source: HRB Open Res. 2020 Apr 20;3:16. [Version 1] doi: 10.12688/hrbopenres.13031.1 (PMC7509595; doi:10.12688/hrbopenres.13031.1)
Supplement: Peer review history

# Peer review: Reviewer response for version 1

**DOI:** 10.21956/hrbopenres.14126.r27908

**Published:** 2020-09-22

**Authors:** Elaine Wethington

**Affiliations:** 1 Department of Human Development, Roybal Center, Cornell University, Ithaca, NY, USA

The authors plan to conduct a longitudinal (2-interview) qualitative interview study about beliefs, behaviors, and experiences in the COVID-19 pandemic with around 30 older Irish adults. The rationale for the study is well-presented and the study is timely in the context of greater susceptibility of severe disease among older adults. The authors will organize the presentation of their findings using the COREQ framework. The presentation of the protocol might be improved with attention to three different areas. First, the authors plan to recruit a heterogenous sample, but do not provide specific recruitment targets to assure that heterogeneity. Second, it would be helpful to provide more information about the coding methods that will be used. Third, the authors stated that a qualitative study is important (at least in part) because it may provide different information than a quantitative study. It may be useful for the authors to plan ahead to compare their findings to quantitative studies currently being conducted about the use of recommended infection prevention measures. The questions may therefore need more directive probing to achieve comparability with other studies.

Is the study design appropriate for the research question?

Yes

Is the rationale for, and objectives of, the study clearly described?

Yes

Are sufficient details of the methods provided to allow replication by others?

Partly

Are the datasets clearly presented in a useable and accessible format?

Not applicable

Reviewer Expertise:

Social isolation, stress processes, and transitions across the life course.

I confirm that I have read this submission and believe that I have an appropriate level of expertise to confirm that it is of an acceptable scientific standard.

# Peer review: Reviewer response for version 1

**DOI:** 10.21956/hrbopenres.14126.r27334

**Published:** 2020-05-29

**Authors:** Krystal Warmoth

**Affiliations:** 1 University of Exeter Medical School, University of Exeter, Exeter, UK

Overall, this qualitative study is timely and potentially very impactful. Very little is known about the experiences of older adults during outbreaks and pandemics. I found this protocol well-written and thoughtful. I only have a few comments that may need some clarifications and expounding.

1. More information about the method adaptations that may need to occur with changing guidance and policies over the course of the study. For example, if the cocooning is lifted before the 4-6 week follow-up interview how will this be handled?
2. Any time that ‘to date’ or ‘currently’ are mentioned, you need to state the date as work and knowledge regarding COVID-19 is quickly evolving and ongoing.
3. More information about your inclusion and exclusion criteria would be valuable.
4. As you have included carers in your sample, you may want to consider how their responses will differ and how you are going to treat and analyse them (possibly separately).
5. You are also not excluding people who had the virus or been in contact with people who have had it. How will you treat the responses from these individuals (e.g., questions asked, data analysed)? It may be interesting to see how these responses differ.
6. More details regarding coding (such as inductive or deductive) and how theme generation will occur would be useful for the reader to understand the analysis.
7. You stated that ‘the interview schedule was developed with attention to the potential psychosocial consequences of the COVID-19 pandemic’. How was this determined? Was any evidence used to inform the schedule? If so, please include in the protocol.

Is the study design appropriate for the research question?

Yes

Is the rationale for, and objectives of, the study clearly described?

Yes

Are sufficient details of the methods provided to allow replication by others?

Partly

Are the datasets clearly presented in a useable and accessible format?

Not applicable

Reviewer Expertise:

NA

I confirm that I have read this submission and believe that I have an appropriate level of expertise to confirm that it is of an acceptable scientific standard.
